# Influence of the Physical State of Spray-Dried Flavonoid-Inulin Microparticles on Oxidative Stability of Lipid Matrices

**DOI:** 10.3390/antiox8110520

**Published:** 2019-10-30

**Authors:** Guibeth Morelo, Begoña Giménez, Gloria Márquez-Ruiz, Francisca Holgado, Patricio Romero-Hasler, Eduardo Soto-Bustamante, Paz Robert

**Affiliations:** 1 Departamento de Ciencia de los Alimentos y Tecnología Química, Facultad de Ciencias Químicas y Farmacéuticas, Universidad de Chile, Santos Dumont 964, Independencia, Santiago 8380494, Chile; guibeliliana@gmail.com; 2 Departamento de Ciencia y Tecnología de los Alimentos, Facultad Tecnológica, Universidad de Santiago de Chile, Av. Ecuador 3769, Estación Central, Santiago 9170124, Chile; bego.gimenez@usach.cl; 3 Instituto de Ciencia y Tecnología de Alimentos y Nutrición (ICTAN-CSIC), 28040 Madrid, Spain; gmarquez@ictan.csic.es (G.M.-R.); f.holgado@ictan.csic.es (F.H.); 4 Departamento de Química Orgánica y Fisicoquímica, Facultad de Ciencias Químicas y Farmacéuticas, Universidad de Chile, Santos Dumont 964, Independencia, Santiago 8380494, Chile; patricio_romero@outlook.com (P.R.-H.); esoto@ciq.uchile.cl (E.S.-B.)

**Keywords:** inulin, flavonoid, microparticles, crystallinity, amorphous, lipid oxidation

## Abstract

The effect of the physical state of flavonoid-inulin microparticles (semi-crystalline/amorphous) on the oxidative stability of lipid matrices was studied. Epicatechin (E) and quercetin (Q) microparticles with inulin were formulated at two infeed temperatures (15 °C and 90 °C) by spray drying. X-ray diffraction analyses showed that flavonoid-inulin microparticles obtained at feed temperature of 15 °C were semi-crystalline (E-In-15, 61.2% and Q-In-15, 60%), whereas those at 90 °C were amorphous (Q-In-90, 1.73 and Q-In-90 2.30%). Semi-crystalline state of flavonoid-inulin microparticles enhanced the EE (68.8 and 67.8% for E and Q, respectively) compared to amorphous state (41.6 and 51.1% for E and Q, respectively). However, amorphous Q-microparticles showed the highest antioxidant activity both in methyl linoleate and sunflower oil, increasing the induction period and decreasing the polar compounds and polymer triglyceride formation during long-term oxidation study. Therefore, the physical state of spray-dried flavonoid-inulin microparticles may determine their antioxidant activity in lipid matrices.

## 1. Introduction

Lipid oxidation is one of the major causes of food deterioration, leading to the loss of nutrients and bioactives, off flavors development, and could also form potentially toxic compounds [1]. The use of synthetic antioxidants, such as butylated hydroxytoluene, butylated hydroxyanisole, or n-propyl gallate to delay lipid oxidation is currently questioned due to their possible harmful effects on human health [2], and the use of natural antioxidants is of growing interest. In recent years, flavonoids have been used to improve the oxidative stability of foods. The main mechanisms by which flavonoids act against lipid oxidation involve transferring a hydrogen atom or electrons to a peroxyl radical and metal chelation [3]. Free flavonoids, mainly quercetin, myricetin, morin, (+)catechin, (−)epigallocatechin, (−)epicatechin, and kaempferol have been incorporated in different lipid systems with varying degrees of success in terms of oxidative stability [4,5,6,7,8,9,10,11,12]. The incorporation of free flavonoids in lipid systems may have some drawbacks since these phenolic compounds have limited solubility and stability [13], together with unpleasant flavors in some cases [14].

Encapsulation of flavonoids is a technology which could enhance the stability of flavonoids. Among the encapsulation methods, spray-drying is the most widely used for the encapsulation of bioactive compounds in the food industry [15]. Inulin is a linear fructo-oligosaccharide composed of fructosyl units (β (2→1) linkage) and usually contains one terminal glucose moiety per molecule. β-linkage makes inulin resistant to hydrolysis by human gastrointestinal enzymes [16] and it is classified as colonic delivery biopolymer [17]. Although inulin is moderately water soluble, it is used as an encapsulating agent by spray drying due to low viscosity and colorless solutions [18]. However, there are few studies focused on the effect of the incorporation of microencapsulated flavonoids on the oxidative stability of lipid systems [19,20]. In previous studies undertaken by our research group, flavonoids were microencapsulated with inulin (lipid-insoluble polymer), where channelizing agents (lipid-soluble polymer) were incorporated. These microparticles were able to release the encapsulated flavonoids and improve the oxidative stability of methyl linoleate [21] and vegetable oils [22], which was attributed to the formation of channels inside the microparticles, favoring the diffusion of flavonoids. 

In general, powders obtained by spray-drying are amorphous. However, in the case of inulin, process variables of spray-drying such as feed temperature and inlet air temperature have been reported to influence both the morphology and the degree of crystallinity of the microparticles [23,24]. When the flavonoid-inulin microparticles are incorporated into a lipid system, the physical state of these microparticles (semi-crystalline/amorphous) may influence the oxidative stability of the system, but this still has not been addressed. 

The objective of this research was to design flavonoids (epicatechin and quercetin) microparticles with inulin as encapsulating agent and to evaluate the influence of the physical state of spray-dried flavonoid microparticles (semi-crystalline/amorphous) on the oxidative stability of lipid systems.

## 2. Materials and Methods 

### 2.1. Material

Inulin (In) Raftilina^®^ HP (DP ≥ 23) was obtained from Alfa-Chilena (Chile), quercetin (Q) ≥ 90%, epicatechin (E) ≥ 90%, methyl linoleate (ML) > 99%, and alpha-tocopherol ≥ 90% were obtained from Sigma-Aldrich (Chile). Sunflower oil (SO) (Natura, AGD Natural Food, Argentine) without synthetic antioxidants was acquired from local market. The major fatty acids were 51.3 ± 0.1% (C18:2 ω6), 37.3 ± 0.1% (C18:1 ω9 cis), 5.73 ± 0.01% (C16:0), and 3.27 ± 0.04% (C18:0). The alpha-tocopherol (AT) content was 548 ± 9 mg/kg oil. The initial values of polar compounds (PC) and peroxide value were 4.20 ± 0.04% and 0.37 ± 0.10 mEq O_2_/kg SO, respectively.

### 2.2. Methods 

#### 2.2.1. Preparation of the Flavonoids Microparticles 

Epicatechin (E) and Quercetin (Q) microparticles with inulin (In) were prepared by spray drying under optimal conditions of inlet air temperature (160 °C) and flavonoid:inulin ratio (1:41 and 1:43, respectively for E-In and Q-In), according to [21]. Inulin (13.94 and 14.62 g for E-In and Q-In, respectively) was dispersed in water (68.85 and 68.34 g, respectively) and heated at 70 °C with constant stirring. Afterwards, the dispersions were cooled at room temperature. Q and E (0.34 g) in ethanol (16.87 and 16.73 g for E-In and Q-In, respectively) were added to the polymer dispersion and stirred for 30 min. Each resultant infeed was fed into a spray-dryer (mini Spray-Dryer B-290, Büchi, Switzerland) at two infeed temperatures, 15 °C and 90 °C (E-In-15, E-In-90, Q-In-15 and Q-In-90). The drying conditions for airflow, rate of feeding, and atomization pressure were 600 L/h, 3 mL/min, and 138 KPa, respectively. The powders obtained were stored in the dark at −20 °C until analysis. Furthermore, empty microparticles (In-15 and In-90) were prepared as control. 

#### 2.2.2. Characterization of Flavonoid Microparticles.

##### X-ray Diffraction Analysis 

Diffractograms were recorded with an X-ray diffractometer (D8-Advance, Bruker, Germany) from 4° to 80° (2θ) at room temperature, using a fixed time of 0.5 s per step of 0.01° and a Cu K_α1_ radiation (40 kV 30 mA) in a Bragg Brentano geometry. The crystallinity indexes were estimated between 4° and 30° in 2θ [25,26] from the ratio of the integrated intensity of the crystalline peaks to the total integrated intensity of coherent scattering after appropriate baseline subtraction [27,28]. The software used for fitting the curves was Diffrac.Suite TOPAS (v4.1; Bruker, Germany).

##### Differential Scanning Calorimetry 

The thermal analyses of the flavonoids (E and Q) and the microparticles (In-15, In-90, E-In-15, E-In-90, Q-In-15, and Q-In-90) were carried out with a Differential Scanning Calorimetry and Thermal Gravimetric Analysis TGA/DSC-2STARe system (Mettler Toledo, Switzerland), using the Analysis Stare software. The microparticle samples (in the range 5 to 10 mg) were placed in ceramic open capsules. Temperature sweeps were run from 25 °C to 300 °C, at a heating and cooling rate of 5 °C/min under a nitrogen flux of 20 mL/min.

##### Encapsulation Efficiency and Recovery of Flavonoids

Surface flavonoids: microparticles (E-In-15, E-In-90, Q-In-15, and Q-In-90) (100 mg) were dispersed in methanol (4 mL) and softly stirred. The supernatant was transferred to a volumetric flask (25 mL). It was then filled up with water:methanol: acetonitrile (45:40:15 v/v/v) containing glacial acetic acid (1% v/v), and an aliquot was injected into the high performance liquid cromatography (HPLC) according to [21]. 

Total flavonoids: microparticles (E-In-15, E-In-90, Q-In-15, and Q-In-90) (100 mg) were treated with 3 mL of water:ethanol:acetone (50:25:25 v/v/v), vortexed for 1 min, ultra-sonicated for 20 min, and centrifuged at 452.8 g for 3 min. The supernatant was removed and the process was repeated twice. The supernatants were transferred to a volumetric flask (50 mL), filled up with water:methanol:acetonitrile (45:40:15 v/v/v) containing glacial acetic acid (1% v/v), and an aliquot was injected into the HPLC according to Palma et al. [21]. 

Encapsulation efficiency (EE) and recovery (R) of flavonoids were calculated according to Equations (1) and (2), respectively:

(1) $$\mathrm{EE}\;\left(\%\right)=\frac{\mathrm{Total}\;\mathrm{flavonoid}\;\left(Q\;\mathrm{or}\;E\right)-\mathrm{Surface}\;\mathrm{flavonoid}\;\left(Q\;\mathrm{or}\;E\right)}{\mathrm{Total}\;\mathrm{flavonoid}\;\left(Q\;\mathrm{or}\;E\right)}\times 100$$

(2) $$R\;\left(\%\right)=\frac{\mathrm{Total}\;\mathrm{flavonoids}\;\left(Q\;\mathrm{or}\;E\right)\;\mathrm{in}\;\mathrm{the}\;\mathrm{powder}\;\left({\mathrm{mg}\;g}^{-1}\right)}{\mathrm{Total}\;\mathrm{flavonoids}\;\left(Q\;\mathrm{or}\;E\right)\;\mathrm{in}\;\mathrm{the}\;\mathrm{feed}\;\mathrm{solution}\;\left({\mathrm{mg}\;g}^{-1}\right)\;}\times 100$$

##### Moisture Content, Hygroscopicity and Water Activity (a_w_)

Water activity (a_w_) was determined by the dewpoint method, using a Hygrolab 2 (Rotronic, USA) at 20 ± 0.3 °C, and moisture was determined according to AOAC [29]. The hygroscopicity was determined according to the procedure described by Cai and Corke [30].

##### Morphology and Particle Size of the Microparticles 

The morphology and outer structure of empty microparticles (In-15 and In-90) and flavonoid microparticles (E-In-15, E-In-90, Q-In-15, and Q-In-90) were evaluated by scanning electron microscopy (SEM). Microparticles were coated with gold/palladium using a Varian Vacuum Evaporator PS 10E and analyzed using a LEO 142 OVP (LEO Electron Microscopy Ltd., Cambridge, UK), operated at 20 kV. The scanned images were collected digitally using EDS 7424 software (Oxford Instruments, Oxford, UK). 

The particle size and size distribution were determined by light scattering using a laser diffraction particle size analyzer (LV 950-v2, HORIBA, Japan) with a lens of 300 mm. Microparticles were dispersed in propylene glycol prior to analysis and the results were expressed as volume average diameter (D_(4,3)_).

#### 2.2.3. Oxidation Assays

##### Rancimat 

The induction period (h) was determined in samples of ML, used as lipid model system devoid of antioxidants, and SO (3 g), with empty microparticles (In-15 and In-90) and flavonoid microparticles (E-In-15, E-In-90, Q-In-15, and Q-In-90) (equivalent amount of 200 mg flavonoid/kg). A Rancimat Oxidative Stability Instrument (Metrohm Ltd., Herisau, Switzerland) was used at 60 °C and air flow of 20 L/h [31]. Effluent air containing volatile organic acids from the oxidized samples was collected in a vessel with distilled water and conductivity measured automatically. The assays were carried out in triplicate. Protection factor (PF) was calculated according to Equation (3).

(3) $$\mathrm{PF}=\frac{\mathrm{Induction}\;\mathrm{period}\;\mathrm{of}\;\mathrm{ML}\;\mathrm{or}\;\mathrm{SO}\;\mathrm{with}\;\mathrm{flavonoid}\;\mathrm{microparticle}\;\mathrm{added}\;}{\mathrm{Induction}\;\mathrm{period}\;\mathrm{of}\;\mathrm{ML}\;\mathrm{or}\;\mathrm{SO}}$$

##### Long-Term Assays 

Open glass tubes (10 cm × 1 cm) with SO (3 g) with empty microparticles (In-15 and In-90) and flavonoid microparticles (E-In-15, E-In-90, Q-In-15, and Q-In-90) were placed in tubes and heated at 60 ± 1 °C in a heating block (Merck, Germany) with constant stirring (350 rpm) for 35 days. Tubes (in triplicate) were removed at specific time intervals (3, 7, 11, 14, 21, 28, and 35 days) to determine polar compounds and alpha-tocopherol content.

• Polar Compounds and Distribution of Polar Compounds

Polar compounds (PC) were determined by adsorption column chromatography [32] and the distribution of PC by high performance size exclusion chromatography (HPSEC) [33]. The separation and quantification of the polar compounds was performed by HPSEC, using a chromatograph equipped with a Waters 510 pump (Waters, Milford, USA), a Rheodyne 7725i injector (10 mL sample loop), and a refractive index detector (HP 1037 A, Agilent Technologies, Palo Alto, USA). The separation was performed on 100 and 500 Å columns (25 cm × 0.77 cm i.d.) packed with porous, highly cross-linked styrene-divinylbenzene copolymers (film thickness 5 mm) (Agilent Technologies, Palo Alto, USA) connected in series, with tetrahydrofuran (1 mL/min) as mobile phase.

• Tocopherols

Tocopherols were determined by HPLC according to AOCS [34]. The HPLC equipment consisted of a Merck-Hitachi L-7110 pump with a Hitachi 5440 fluorescence detector, a LiChroCART Superspher Si-60 column (5 μm particle size, 4 mm i.d. × 250 mm; Merck, Darmstadt, Germany), and a 20 μL injection loop. Propan-2-ol:n-hexane (1:99 v/v) was used as mobile phase at a flow rate of 1 mL/min. Detection was performed at 290 nm and 330 nm of excitation and emission wavelengths, respectively. The alpha-tocopherol content was quantified using an external standard. 

#### 2.2.4. Statistical Analysis

All the experiments were performed in triplicate. ANOVA one-way was applied to determine the statistical differences among systems using Statgraphics, Version 7.0 (Manugistic Inc., Statistical Graphics Corporation, Rockville, MD, USA).

## 3. Results

### 3.1. Characterization of the Microparticles

#### 3.1.1. X-ray Diffraction Analysis 

**Figure 1:**
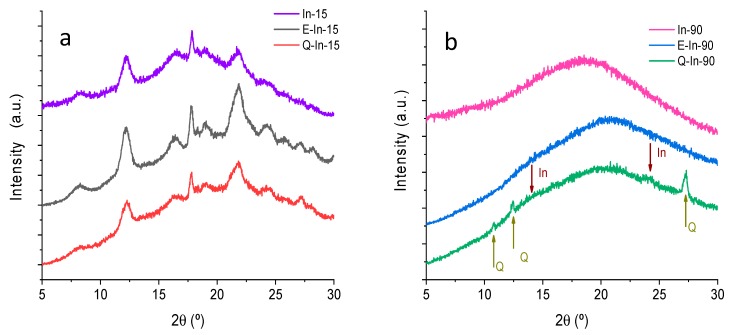
X-ray diffractograms for In-15, E-In-15, and Q-In-15 (**a**), and for In-90, E-In-90, and Q-In-90 (**b**).

Figure 1 shows the X-ray diffractograms performed on empty microparticles (In-15 and In-90) and flavonoid microparticles (E-In-15, E-In-90, Q-In-15, and Q-In-90). The empty microparticles (In-15 in Figure 1a, and In-90 in Figure 1b) showed either a broad scattering halo or the same halo with superimposed sharp diffraction peaks, which is typical for an amorphous and semi-crystalline material, respectively. Based on the diffraction patterns obtained for In-15, the orthorhombic monohydrated crystalline form of inulin was identified [35]. According to Ronkart et al. [23], the absence of the 2θ = 10.62° diffraction peak discarded the existence of the pseudo-hexagonal semi-hydrated form. As expected, In-90 showed a broad amorphous behavior, while In-15 had characteristic peaks of a semi-crystalline material [25,26]. The crystallinity index for the In-90 and In-15 was 18.6% and 55.8%, respectively. 

The incorporation of flavonoids into the inulin microparticles (17.2–19.5 mg/g powder) obtained at both feed temperatures (15 °C and 90 °C), did not noticeably modify the diffraction patterns. At the highest feed temperature, Q-In-90 and E-In-90 were mostly amorphous (Figure 1b), while at the lowest feed temperature, Q-In-15 and E-In-15 were semi-crystalline (Figure 1a). The microparticles with flavonoids prepared at feed temperature of 15 °C (E-In-15 and Q-In-15) showed a degree of crystallinity of 61.2% and 60.0%, respectively, similar to empty In-15 microparticles (55.8%). The incorporation of the flavonoids to the In-90 microparticles significantly decreased the degree of crystallinity with respect to the empty In-90 microparticles (18.6%), showing crystallinity indexes of 1.73% and 2.3% for E-In-90 and Q-In-90, respectively. This decrease suggests that the E and Q were encapsulated or dispersed in the polymer matrix at 90 °C, forming a more amorphous complex, where intermolecular interactions between flavonoids and inulin take place by hydrogen bonds. This result has been also reported for Q nanoparticles using nanoprecipitation with polyvinyl alcohol as encapsulating agent (Wu et al., 2008), and in microparticles of Q with alginate and chitosan [36].

The sharper reflexes found at 18.15° and 24.96° in 2θ in E diffractograms and at 12.47° and 27.47° in Q diffractograms should be easily observed in the amorphous E-In-90 and Q-In-90 microparticles, in spite of the low flavonoid load in the microparticles (17.2–19.5 mg/g powder). However, peaks reflecting the crystalline character of E were not found either in E-In-90 or in E-In-15 microparticles. In the case of Q-In-90, two sharp reflexes were found at 12.42° and 27.71° in 2θ. These two peaks were also detected in Q-In-15, but overlapped with the crystalline peaks of inulin. Therefore, this result suggests that E was homogeneously dispersed in the inulin polymer matrix, whereas Q segregated in crystalline domains.

Considering the diffractograms obtained for all the samples and the quantitative determination of the crystallinity index, it was possible to obtain microparticles with different degrees of crystallinity. Therefore, the selection of the drying parameters, such as the feeding temperature, provides a strategy to set the degree of crystallinity of the spray-dried powders.

#### 3.1.2. Thermal Analysis (TGA/DSC)

The DSC curve of E showed a sharp endothermic peak at 246.43 °C (ΔHf = 156.9 J/g), caused by the melting of E (Figure S1). These results agree with the reported melting of E [37]. TGA analysis showed that the thermal degradation of this flavonoid began at the melting temperature. In the case of Q, two endothermic peaks by heating were well distinguished at 99.73 °C and 322.02 °C. Both peaks have been described by Moreira da Costa, Barbosa Filho, Gomes do Nascimento, & Oliveira Macedo [38]. The first one (99.73 °C) corresponded to the water loss, and the second one (322.02 °C) was caused by the melting of Q, which immediately decomposes in the isotropic state. These two endothermic peaks were confirmed by the TGA results (Figure S1). 

**Figure 2:**
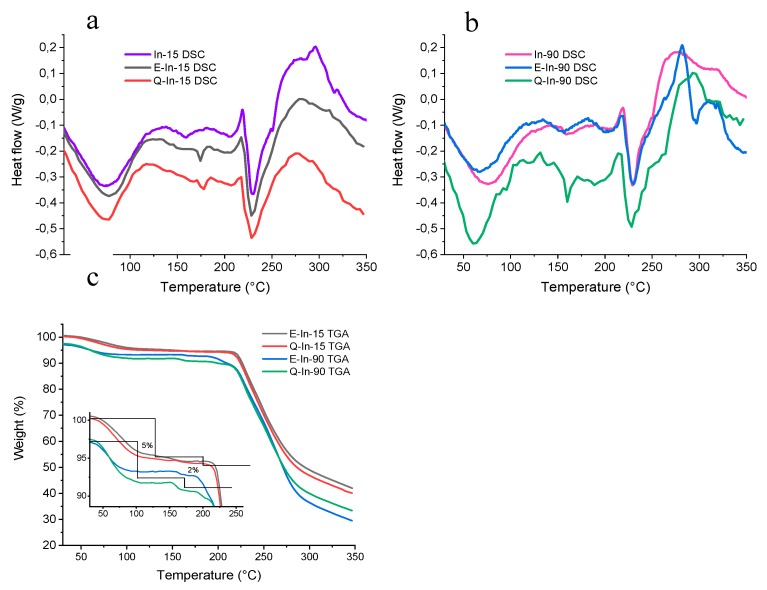
DSC (**a**,**b**) and TGA (**c**) curves for empty microparticles (In-15 and In-90) and flavonoid-inulin microparticles (E-In-15, E-In-90, Q-In-15, and Q-In-90).

For the empty microparticles (In-15 and In-90), the DSC curves (Figure 2a,b) were similar to those described by Ronkart et al. [23] as for water loss, melting behavior, and degradation. For all the studied samples, the glass transitions were hard to identify in the thermograms and they were not reported. The water loss occurred between 25 °C and 125 °C. Afterwards, two endothermic peaks were found at around 156 °C and 201 °C. Finally, melting occurred at 230 °C for both samples, followed by material decomposition. Hébette et al. [39] reported the formation of different types of crystals during the crystallization of inulin solutions. Furthermore, the crystallization of inulin resulted into different lamellar structures, where the crystals were separated by amorphous regions, explaining the existence of three to four endothermic peaks. On the other hand, Ronkart et al. [23] reported the existence of these two endothermic peaks, at similar temperatures to those found in this study, which corresponded with the presence of two crystals populations. Therefore, these peaks found at 156 °C and 201 °C may correspond to two different crystal populations, which mostly differ in the polymerization degree [23].

The thermal behavior of flavonoid microparticles (E-in 15, E-In 90, Q-In 15, and Q-In 90) were comparable to that of empty microparticles (Figure 2a,b). However, a new peak around 175 °C appeared in this case for all the flavonoid microparticles, which may be explained as a structural modification of inulin due to the presence of flavonoids. This peak may be a modification of the crystalline structure that melts at 156 °C, corresponding to the lowest polymerization degrees. Therefore, there was a clear preference for flavonoid to interact with the less thermally stable inulin structure.

Figure 2c shows the TGAs of the flavonoid microparticles. The amorphous microparticles (E-In-90 and Q-In-90) showed a lower initial mass than the semi-crystalline ones (E-In-15 and Q-In-15), in agreement with the suggested difference in the water mobility inside the material proposed by Ronkart et al. [25]. An initial loss of 5% in mass was observed in all the thermograms, corresponding to the dehydration of inulin from mono-hydrate to hemi-hydrate. Subsequently, a further mass loss of approximately 2% was found in the range 160 to 180 °C. This last process may be associated with the two melting peaks previously reported in the DSC (Figure 1) in the same temperature range. These losses should be related to residual water which is present in the more stable hemihydrate forms that melt at 156 °C and 201 °C. Ronkart et al. [25] attributed such small water loss to water evaporation and confirmed this supposition by thermogravimetry coupled to a mass spectrometer. Then at this point we can assume a second loss of hydration water now from the hemihydrate polymorph crystals during their melting.

Finally, the flavonoid microparticles lost a large amount of weight from about 200 to 225 °C because of the thermal decomposition of inulin, forming brownish di-D-fructose dianhydrides due to the breakdown of the fructose chains [40].

#### 3.1.3. Encapsulation Efficiency and Recovery of Flavonoids

Table 1 shows the characterization of both empty (In-15 and In-90) and flavonoid microparticles with In (E-In-15, E-In-90, Q-In-15, and Q-In-90). EE represents the interaction between inulin and flavonoids, mainly by hydrogen bonds. EE for E and Q was significantly higher in those microparticles obtained with lower feed temperature, showing that the semi-crystalline state of the microparticles enhanced the EE of both flavonoids compared to the amorphous state. The structural features of the encapsulated flavonoid have been reported to affect the EE values [21,22]. However, Q and E showed significant differences in EE only in the case of the amorphous microparticles in this study.

Recovery of flavonoids represents the loss of flavonoids during the spray drying process. All the systems showed high R values above 75%, which may be attributed to the rapid formation of the crust around the droplets and/or the short residence time in the drying chamber, since the microparticles reach high temperatures for very short periods of time [15]. 

#### 3.1.4. Moisture Content, Hygroscopicity, and a_w_

With regard to moisture content of the microparticles, the amorphous microparticles (E-In-90 and Q-In-90) showed higher moisture content and hygroscopicity than semi-crystalline ones (E-In-15 and Q-In-15) (Table 1). It is known that amorphous powders are more hygroscopic than their crystalline counterparts, which may affect the microparticle stability during storage [24]. The a_w_ found in the microparticles systems was in the range 0.13–0.27, ensuring the microbial stability of the powders.

#### 3.1.5. Morphology and Particle Size of the Microparticles

**Figure 3:**
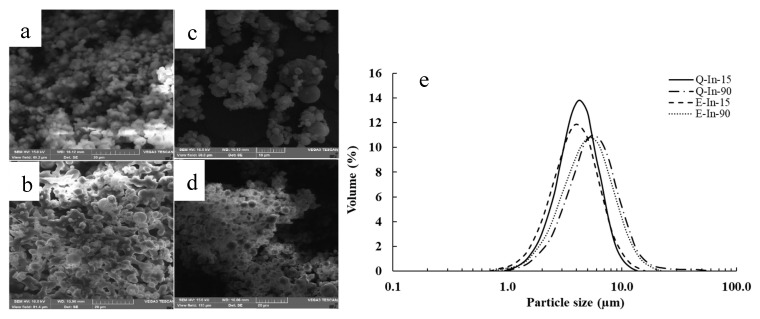
SEM photographs (**a**–**d**) and particle size distribution (**e**) for flavonoid-inulin microparticles. (**a**): E-In-15; (**b**): E-In-90; (**c**): Q-In-15; (**d**): Q-In-90.

Figure 3a–d shows the SEM photographs and particle size distribution of the microparticles powders. The systems with semi-crystalline structure (E-In-15 and Q-In-15) showed microparticles with spherical shape and predominantly smooth surface, although some microparticles showed rough surfaces. In contrast, the amorphous systems (E-In-90 and Q-In-90) consisted in microparticles with irregular shape and highly agglomerated. The presence of agglomerations may result from the collision among particles during drying and/or the high feed temperatures, where single droplets from solubilized feed may join together [24,41]. The distribution of particle size was unimodal in all the microparticles systems (Figure 3e), and the powder particle size, evaluated by D_4,3_, was significantly higher in the systems with amorphous structure than in the corresponding semi-crystalline systems (Table 1).

### 3.2. Oxidation Assays

#### 3.2.1. Rancimat

Table 2 shows the induction period (IP) and protection factor (PF) values of ML and SO without and with flavonoid microparticles (semi-crystalline and amorphous; equivalent amount of 200 mg flavonoid/kg of ML or SO) evaluated in Rancimat at 60 °C. 

The addition of both Q-microparticles (Q-In-15 and Q-In-90) and E-In-90 microparticles to ML gave a significant increase of IP values with respect to ML-control. Furthermore, Q-microparticles (Q-In-15 and Q-In-90) showed significantly higher IPs than E-microparticles, and the highest value was found for amorphous Q-microparticles (Q-In-90). However, the IP of ML was slightly but not significantly higher in the amorphous E-microparticles (E-In-90) than in the semi-crystalline ones (E-In-15). These results suggest that the physical state of the flavonoid microparticles (semi-crystalline/amorphous) could influence the antioxidant activity of the encapsulated flavonoids, thus the oxidative stability of ML. The amorphous state of the flavonoid microparticles would increase the contact surface area between the microparticles and ML, favoring the diffusion of the flavonoids to ML. Furthermore, a higher molecular mobility has been reported in amorphous microparticles [21], which may also improve the diffusion of flavonoids. Another important factor to consider is the structural features of the flavonoid. Thus, E has higher polarity than Q [42], which may impair its diffusion to the lipid medium, explaining the lower IP values obtained for E-microparticles. Furthermore, in spite of Q and E have the same number of phenolic hydroxyl groups, Q also has a double bond in C2-C3 of ring C and a carbonyl group in 4 of ring C, allowing the stabilization of the resonant phenoxyl radical, and therefore, the antioxidant activity [43].

The IP values were higher in SO than in ML, which is expected due to the content of alpha-tocopherol in SO, acting as antioxidant [44]. The IP values of SO significantly increased when Q- and E-microparticles were added. In the case of Q-microparticles, Q-In-90 (amorphous) gave higher IP values than Q-In-15 (semi-crystalline) microparticles, which is in line with the results found in ML where the amorphous microparticles showed the highest IP value. Contrary, the physical state of E-microparticles did not affect the oxidative stability of SO, and no significant differences were found between E-In-15 and E-In-90, despite SO having a higher polarity than ML. In a previous study, Q-In microparticles also showed a higher antioxidant efficiency than E-In microparticles when they were added to both ML and SO [22].

#### 3.2.2. Long-Term Oxidation Study

**Figure 4:**
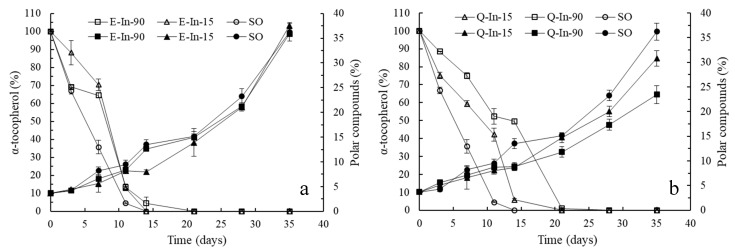
Evolution of polar compounds formation and α-tocopherol loss for SO and SO with flavonoid microparticles (**a**): E-In-15 and E-In-90; (**b**): Q-In-15 and Q-In-90).

Figure 4 shows the evolution of PC formation and α-tocopherol loss for the systems with and without the addition of flavonoid microparticles during the storage at 60 °C for 35 days. Although the PC increased during the storage time in all the samples (*p* < 0.05), those systems with Q showed PC values significantly lower than SO and SO with E-microparticles at day 35. Furthermore, SO systems with Q-In-90 (amorphous) showed PC values significantly lower than SO-Q-In15 (semi-crystalline) (23.5 ± 1.8% and 30.8 ± 1.6% for Q-In-90 and Q-In-15, respectively). These results agree to the induction period in ML and SO, where the physical state of the microparticles and/or the structural flavonoid features influence the release of the flavonoids from the microparticles to oil and, therefore, the oil oxidative stability. The retention of α-tocopherol decreased in SO during the storage time, with and without flavonoid-microparticles (Figure 4). The complete depletion of α-tocopherol marked the induction time, as reported by Martín-Polvillo et al. [43]. The α-tocopherol degradation rate constant was significantly higher in SO-control (17.3 ± 0.4 days^−1^) than in those systems with E-microparticles (16.9 ± 0.01 days^−1^ for E-In-15 and 16.6 ± 0.06 days^−1^ for E-In-90) and Q-microparticles (15.6 ± 0.01 days^−1^ for Q-In-15 and 14.3 ± 0.17 days^−1^ for Q-In-90), suggesting an interaction between α-tocopherol and flavonoids, where α-tocopherol was regenerated by flavonoids [4,5,45]. The tocopherols regeneration reaction by flavonoids has also been reported for quercetin, myricetin, and rutin in ML [5], and quercetin and catechin in ML [4]. This mechanism has been attributed to lower redox-potential of flavonoids, favoring the hydrogen transference to tocopheroxyl radical [46,47]. Furthermore, the degradation rate constant of α-tocopherol was significantly lower in the system with the amorphous state of Q-microparticles compared with the semi-crystalline state of Q-microparticles, whereas the physical state of E-microparticles did not affect the α-tocopherol degradation rate constant. 

Table 3 shows the distribution of PC in different of groups of oxidation compounds and hydrolysis products at day 35 of storage in the systems with and without the addition of flavonoid microparticles. Given the absence of water, hydrolysis reactions can be negligible, and DG and FFA contents were low and similar in all the systems (1.0–1.22% for DG; 0.20–0.33% for FFA). The entire oxidation process may be measured by the quantification of oxidized triacylglycerol monomers (OxTM), triacylglycerol dimers (TD) and polymers (TP) [48]. While OxTM include hydroperoxides formed in the early stage of oxidation, TD and TP start increasing when oxidation is accelerated. The incorporation of flavonoid microparticles did not affect the percentage of OxTM, whereas the formation of both TD and TP was only significantly lower in those systems with Q-microparticles, with the lowest values in the case of amorphous Q-microparticles. Therefore, Q-microparticles, especially in the amorphous state, may act as a polymerization inhibitor during the thermal oxidation of SO. Q has shown an antipolymerization effect during thermal oxidation in bulk ML [49].

## 4. Conclusions

Spray-dried flavonoid-inulin microparticles were designed as antioxidants for lipid systems. The physical state of the flavonoid-inulin microparticles affected the flavonoid-inulin interactions, evaluated as encapsulation efficiency. Semi-crystalline inulin formed more ordered networks, increasing the EE of the flavonoids. However, EE was not in line with the oxidative stability of the lipid systems where the flavonoid microparticles were incorporated. In fact, amorphous state of the flavonoid-inulin microparticles increased the oxidative stability of lipid systems to a greater extent than semi-crystalline state. In addition to the physical state of flavonoid-inulin microparticles, other parameters such as the structural features and solubility of the flavonoids can influence the oxidative stability of the lipid matrices.

## Figures and Tables

**Table 1:** Characterization of empty microparticles and flavonoid microparticles at two infeed temperatures (15 °C and 90 °C).

Parameters	In-15	In-90	E-In-15	E-In-90	Q-In-15	Q-In-90
F/In ratio	-	-	1:41	1:41	1:43	1:43
Infeed temperature (°C)	15 ± 1	90 ± 2	15 ± 1	90 ± 2	15 ± 1	90 ± 2
Inlet temperature (°C)	160	160	160	160	160	160
Crystallinity index (%)	55.7	18.6	61.2	1.73	60.0	2.30
EE Flavonoids (%)	-	-	68.8 ± 1.0 ^c^	41.6 ± 0.7 ^a^	67.8 ± 1.9 ^c^	51.1 ± 2.8 ^b^
R Flavonoids (%)	-	-	82.0 ± 2.0 ^c^	79.1 ± 0.5 ^b,c^	75.7 ± 1.9 ^a^	78.1 ± 1.0 ^b^
Total flavonoids (mg/g)	-	-	19.5 ± 0.48 ^b^	18.8 ± 0.1 ^b^	17.2 ± 0.1 ^a^	17.7 ± 0.2 ^a^
Moisture (%)	5.7 ± 0.3 ^a^	8.5 ± 0.1 ^c^	6.2 ± 0.1 ^a^	7.2 ± 0.1 ^b^	6.3 ± 0.1 ^a^	7.0 ± 0.3 ^b^
Water activity (a_w_)	0.15 ± 0.03 ^a^	0.13 ± 0.0 ^a^	0.22 ± 0.1 ^b^	0.27 ± 0.01 ^c^	0.27 ± 0.1 ^c^	0.42 ± 0.1 ^d^
Hygroscopicity (g/100g)	25.0 ± 0.1 ^a^	46.7 ± 1.6 ^b^	25.4 ± 0.2 ^a^	47.0 ± 0.2 ^b^	24.9 ± 0.5 ^a^	47.4 ± 0.5 ^b^
D_(4,3)_ (μm)	-	-	4.4 ± 0.01 ^a^	5.6 ± 0.05 ^b^	4.4 ± 0.03 ^a^	6.1 ± 0.4 ^c^

E: epicatechin; Q: quercetin; In: inulin; EE: encapsulation efficiency; R: recovery. Different letters in the same raw mean significant differences (*p* ≤ 0.05) among microparticle systems.

**Table 2:** Induction period (IP) and protection factor (PF) for methyl linoleate (ML) and sunflower oil (SO) with and without amorphous or semi-crystalline flavonoid microparticles (200 mg/kg ML or SO).

Systems	Induction Period (IP) (h)	Protection Factor (PF)
ML	10.7 ± 0.2 ^a^	-
ML+E-In-15	12.0 ± 1.6 ^a,b^	1.12
ML+E-In-90	12.7 ± 0.1 ^b^	1.19
ML+Q-In-15	97.5 ± 2.2 ^c^	9.11
ML+Q-In-90	144.1 ± 1.5 ^d^	13.4
SO	175.3 ± 6.6 ^a^	-
SO+E-In-15	225.4 ± 4.3 ^b^	1.28
SO+E-In-90	235.6 ± 4.4 ^b,c^	1.34
SO+Q-In-15	249.2 ± 7.3 ^c^	1.42
SO+Q-In-90	294.0 ± 1.6 ^d^	1.68

Different letters in ML or SO mean significant differences (*p* ≤ 0.05).

**Table 3:** Distribution of polar compounds (PC) for SO with and without amorphous or semi-crystalline flavonoid microparticles (200 mg/kg SO), at day 0 and after 35 days of storage at 60 °C.

Systems	Days	OxTM (%)	TD (%)	TP (%)	DG (%)	FFA (%)
SO	0	3.0 ± 1.0 ^a,b^	0.2 ± 0.06 ^a^	0.0 ± 0.01 ^a^	0.9 ± 0.42 ^a^	0.2 ± 0.12 ^a^
SO+E-In-15	0	3.0 ± 0.03 ^a,b^	0.2 ± 0.01 ^a,b^	0.04 ± 0.01 ^a,b^	0.9 ± 0.05 ^a^	0.2± 0.01 ^a^
SO+E-In-90	0	2.5 ± 0.01 ^a^	0.2 ± 0.05 ^a,b^	0.1 ± 0.01 ^a^	1.1 ± 0.07 ^a^	0.2 ± 0.02 ^a^
SO+Q-In-15	0	3.2 ± 0.1 ^a,b^	0.3 ± 0.01 ^b^	0.1 ± 0.01 ^b^	1.1 ± 0.1 ^a^	0.3 ± 0.01 ^a^
SO+Q-In-90	0	3.8 ± 0.6 ^a,b^	0.3 ± 0.06 ^b^	0.1 ± 0.02 ^a,b^	1.2 ± 0.28 ^a^	0.3 ± 0.1 ^a^
SO	35	16.3 ± 0.6 ^a^	10.1 ± 0.4 ^b^	8.2 ± 0.3 ^b^	1.22 ± 0.4 ^a^	0.33 ± 0.0 ^a^
SO+E-In-15	35	14.4 ± 0.5 ^a^	9.9 ± 0.07 ^b^	10.8 ± 0.1 ^d^	1.0 ± 0.02 ^a^	0.2 ± 0.01 ^a^
SO+E-In-90	35	16.7 ± 0.8 ^a^	9.8 ± 0.2 ^b^	8.0 ± 1.2 ^c^	1.1 ± 0.06 ^a^	0.3 ± 0.05 ^a^
SO+Q-In-15	35	16.5 ± 1.5 ^a^	7.7 ± 1.6 ^b^	5.3 ± 1.6 ^b^	1.0 ± 0.05 ^a^	0.3 ± 0.12 ^a^
SO+Q-In-90	35	15.8 ± 2.0 ^a^	4.4 ± 2.1 ^a^	1.9 ± 1.0 ^a^	1.0 ± 0.1 ^a^	0.3 ± 0.10 ^a^

Different letters in the column SO at day 0 mean significant differences (*p* ≤ 0.05) among microparticle systems. Different letters in the column SO at day 35 mean significant differences (*p* ≤ 0.05) among microparticles systems.

## Supplementary Materials

The following are available online at https://www.mdpi.com/2076-3921/8/11/520/s1, Figure S1: DSC y TGA curves for epicatechin (E) and quercetin (Q).
