# Supplementary figures and images for: Influence of the Physical State of Spray-Dried Flavonoid-Inulin Microparticles on Oxidative Stability of Lipid Matrices
Source: Antioxidants (Basel). 2019 Oct 30;8(11):520. doi: 10.3390/antiox8110520 (PMC6912732; doi:10.3390/antiox8110520)

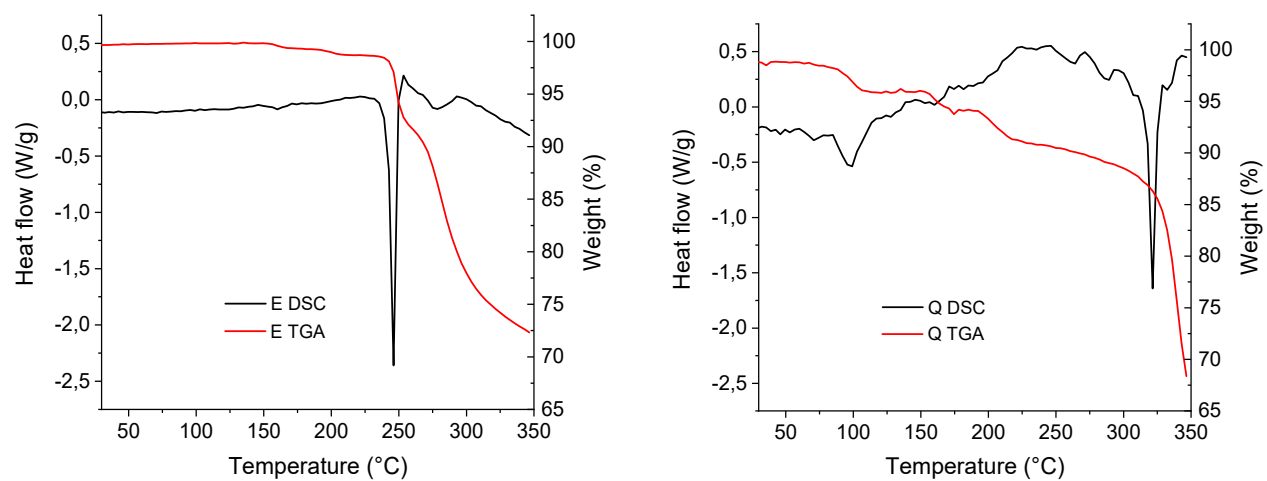

**Figure 1.** DSC y TGA curves for epicatechin (E) and quercetin (Q).

Supplement: Supplementary file 1 [file antioxidants-08-00520-s001.pdf]
